# Supplementary material for: Movie viewing elicits rich and reliable brain state dynamics
Source: Nat Commun. 2020 Oct 5;11:5004. doi: 10.1038/s41467-020-18717-w (PMC7536385; doi:10.1038/s41467-020-18717-w)
Supplement: Supplementary file 1 — Supplementary Information [file 41467_2020_18717_MOESM1_ESM.pdf]

Supplementary Online Content

Movie viewing elicits rich and reliable brain state dynamics

van der Meer et al.

Supplementary Note 1

Movie Details

The Butterfly Circus is a short movie set in the 1930's depression-era rural USA. The movie can be viewed at: [https://www.youtube.com/watch?v=p98KAEif3bI]. Further details of the movie can be found on the internet movie database (IMDB) [https://www.imdb.com/title/tt1507355/] and on Wikipedia: [https://en.wikipedia.org/wiki/The_Butterfly_Circus].

Software Functionality

The scripts used to run all analyses are provided in the Github repository: [https://github.com/brain-modelling-group/MovieBrainDynamics]

A full description of the functionality, installation and user instructions can be found in this public repository; see the readme.md file.

Supplementary Figures

Supplementary Figure 1

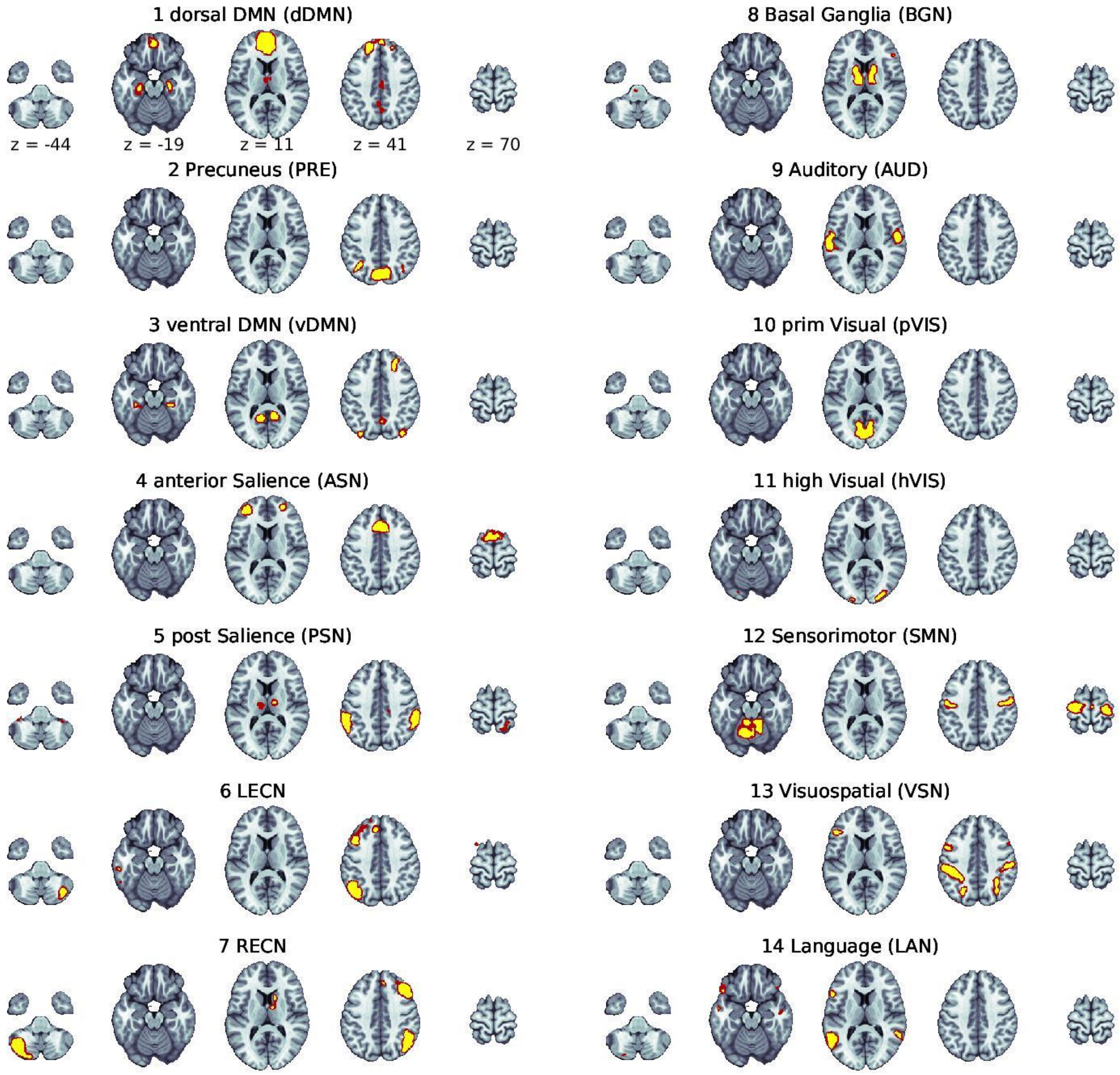

Supplementary Figure 1: The 14 Canonical Brain Networks used to quantify the spatial extent and functional significance of Brain State expression. *These 14 networks are reported in Shirer et al [1] as having high functional inter-connectivity across a wide range of cognitive tasks. The average BOLD signal was extracted from these brain regions, and further preprocessed and analysed according to the steps outlined in the Hidden Markov Model (HMM) methods section.*

Supplementary Figure 2

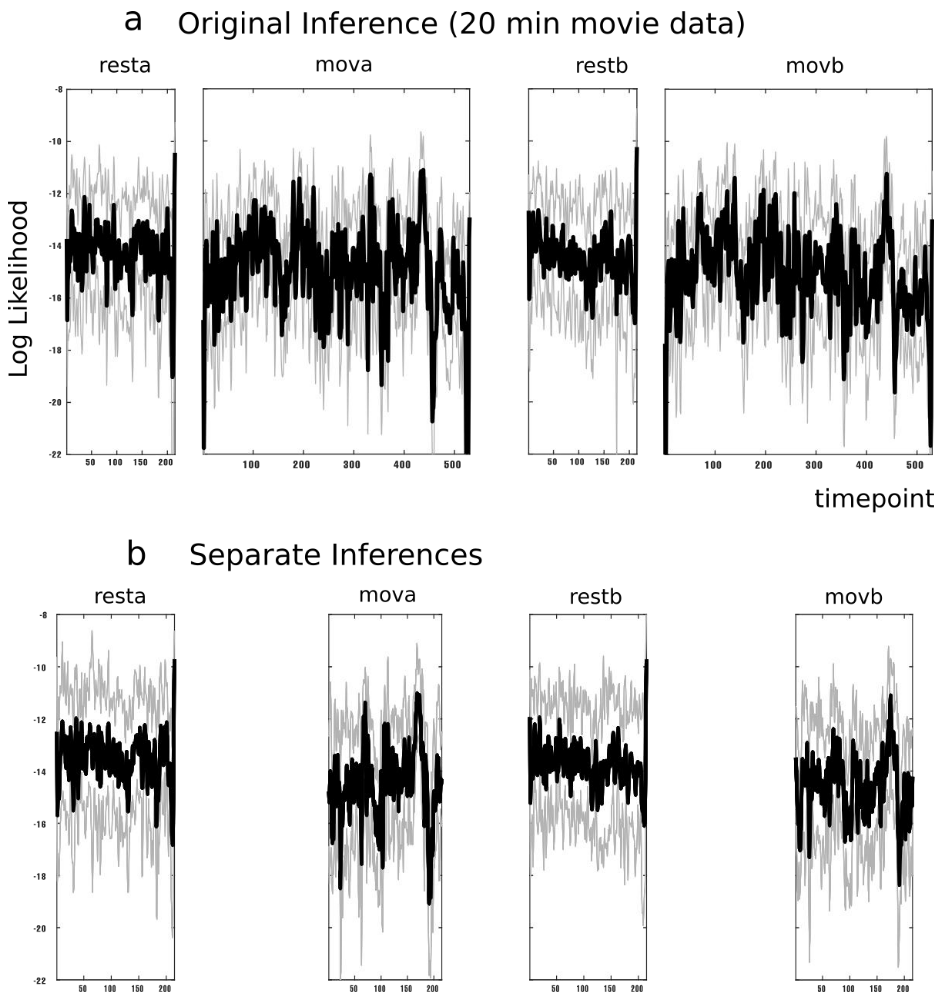

Supplementary Figure 2. Time-resolved HMM log-likelihood, for both rests and movie data. *Grey lines indicate the 95% within-subject confidence intervals (calculated using the optimised Cousineau-Morey method*[2]*).* ***a*** *Results obtained by fitting the HMM on 8 minutes rest and 20 minutes of movie data (original analysis presented in the main text).* ***b*** *Results from independent HMM inversion performed using 8 minutes of resting-state data and 8 minutes of movie data.*

Supplementary Figure 3

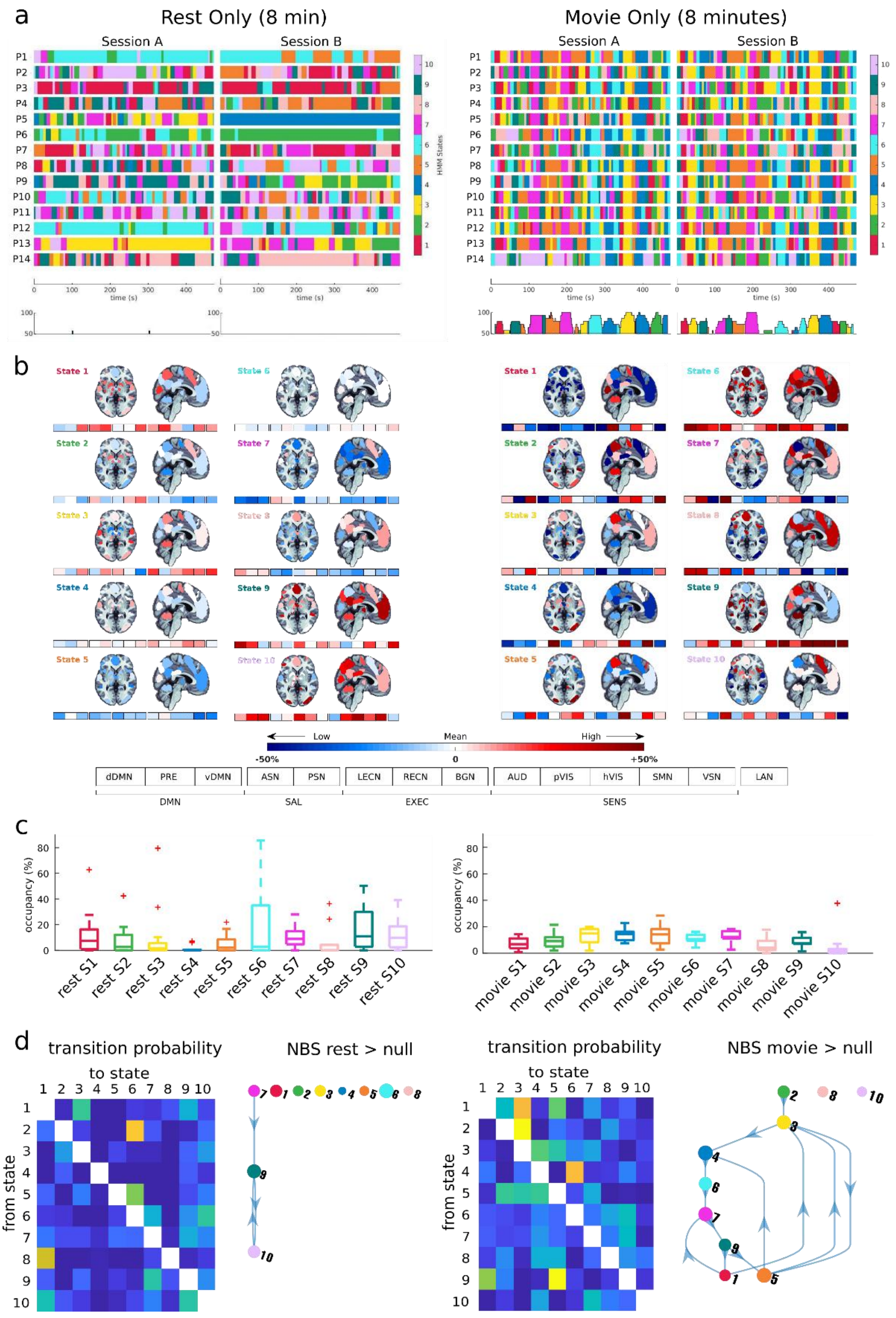

Supplementary Figure 3. Results from distinct HMM inversions computed on 8 minutes of resting-state and 8 minutes of movie data (two sessions, N = 14 participants). *In order to detect the most representative HMM inference among the 15 inversions, we used the Hungarian method[3,4] on the emission probabilities to pair the brain states of each inversion with those of all other inversions. This procedure resulted in 14 values (Pearson's correlations) per inversion, which were then averaged. The*

*inversion with the highest average correlation value was considered as the most representative and used for subsequent analyses. In all panels, brain states are colour-coded according to the legends in panel **b**. **a:** Viterbi State paths for each participant and temporal consistency across participants (between 50 and 100%; bottom trace). **b:** The relative fMRI signal weight onto the 14 canonical networks considered: dorsal and ventral Default Mode Networks (dDMN and vDMN), Precuneus, Anterior Salience Network (ASN), Posterior Salience Network (PSN), Left and Right Executive Control Networks (lECN and rECN), Basal Ganglia Network (BGN), Auditory Network (AUD), Primary Visual Network (pVIS), High Visual Network (hVIS), Sensorimotor Network (SMN), Visuospatial Network (VSN), and Language Network (LAN). These are divided into four main groups: DMN (Default Mode Network); SAL (Salience Network); EXEC (Executive Network) and SENS (Sensory Network). **c:** fractional occupancy; no statistical testing was performed between rest and movie conditions because left/right panels contain separate HMM inversions and there is no one-on-one correspondence between states. Boxplots: upper (lower) box edge: 25th (75th) percentile; central line: median; dotted lines: 1.5 x interquartile length; whiskers extend to the most extreme data points not considered outliers; red plus: outliers. **d:** transition probability matrix (left), and state transitions identified by the Network-Based Statistics ($p_{FWE} < 0.05$; one-sample t-test vs null) (right).*

Supplementary Figure 4

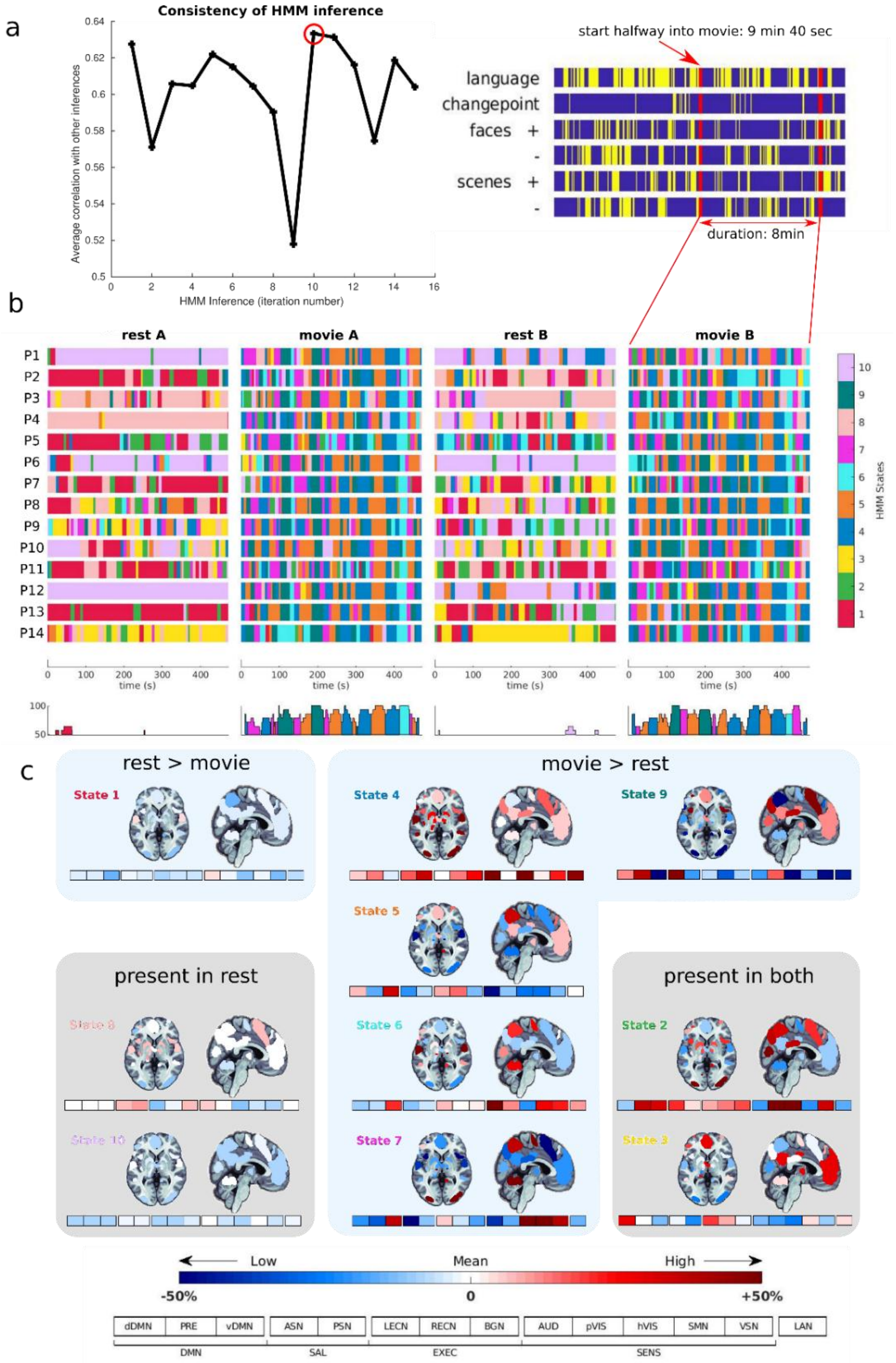

***Supplementary Figure 4. a: HMM inversions on 8 minute concatenated rest and movie data** We estimated the most representative HMM inversion between a total of 15 inversions using the Hungarian method (see the legend of Supplementary Figure 3 for details). **b:** Brain state dynamics during 8 minutes rest and movie viewing, for each participant (raw) and session (top/bottom). The brain states color-coding is associated to the topology of the state presented in panel **c**. As per Fig. 1,*

the temporal consistency across participants, which vary between 50% (chance for the resting state) and 100% (complete consistency) is presented in the bottom panel of each experimental session. ***c:*** *fMRI signal profiles for each of the 10 brain states occurring during resting state and movie viewing. Consistent with results from the primary analyses (Fig. 1), the relative weighting of each brain state onto each of the 14 canonical networks is considered. The blue-red colour bar indicates the relative loading to the average brain states activity.*

Supplementary Figure 5

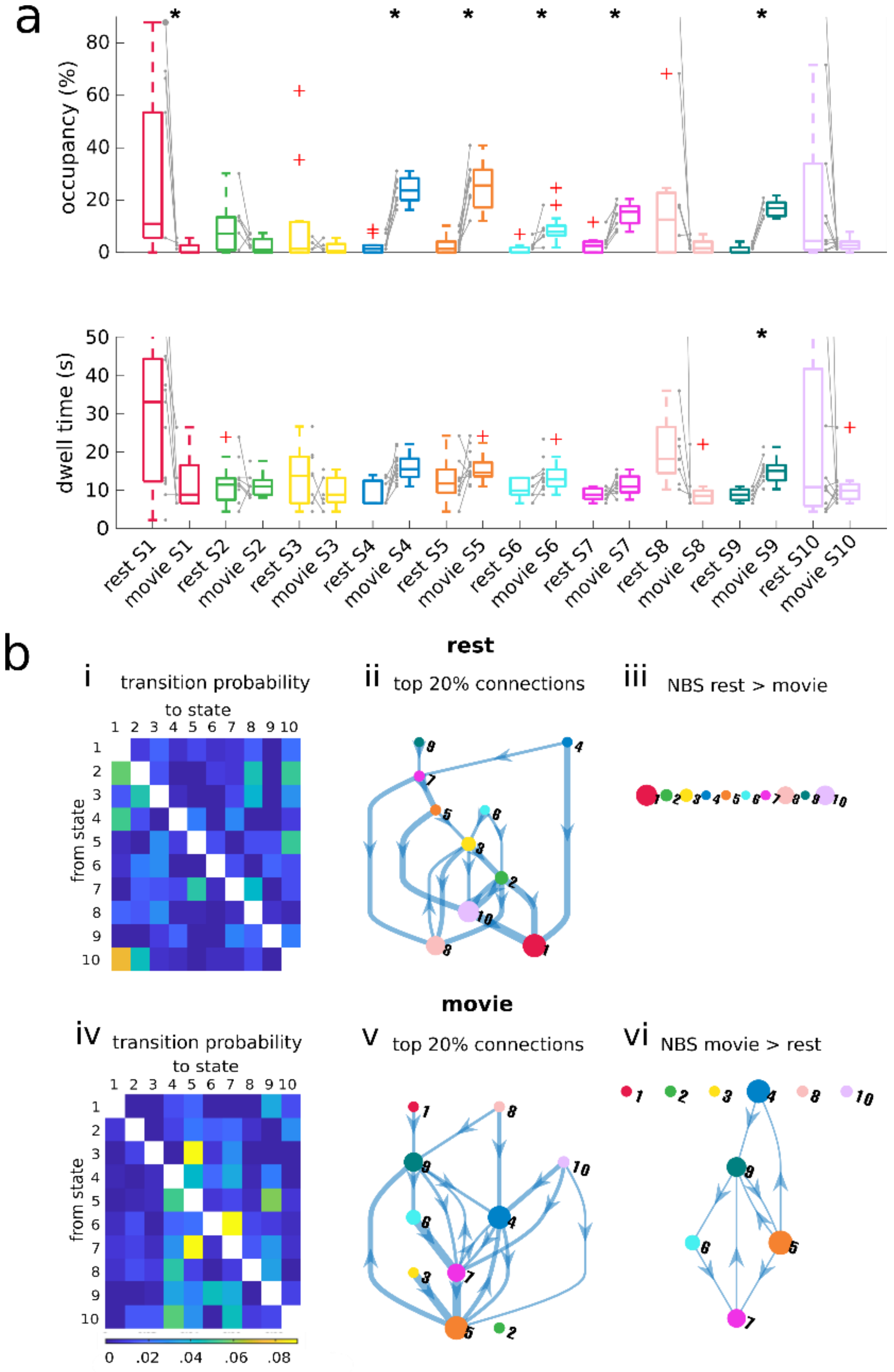

Supplementary Figure 5. Dynamic characteristics of brain states across rest and movie viewing for HMM inversion on concatenated rest and movie sessions of 8 minutes. *Asterisks indicate statistical significance (paired two-sided t-tests, $p < 0.05$; corrected for multiple comparisons across 10 states; n=14 participants examined over 2 consecutive sessions: rest session A and movie session A).* ***a:***

fractional occupancy (FO) and brain state dwell times. Grey lines show how FO and dwell times are paired within participants. The exact p-values for states S1-S10 are: $3.9e^{-3}$; $1.3e^{-2}$; $1.3e^{-1}$; $6.2e^{-9}$; $1.9e^{-7}$; $3.6e^{-4}$; $2.6e^{-7}$; $3.5e^{-2}$; $9.1e^{-11}$; $5.3e^{-2}$. The p-value for difference in dwell time of S9 is $3.0e^{-3}$. Boxplots: upper (lower) box edge: 25th (75th) percentile; central line: median; dotted lines: 1.5 x interquartile length; whiskers extend to the most extreme data points not considered outliers; red plus: outliers. ***b****: brain state transition probabilities during 8 minutes rest and 8 minutes movie viewing. Left column: Group averaged transition probability matrices for rest (i) and movie viewing (iv). Diagonal elements are omitted. Middle column: Top 20% state transitions during rest (ii) and movie viewing (v). Arrow thickness corresponds to the group-averaged probability of that transition. Right column: State transitions having a significantly higher probability of occurring during rest than during movie viewing (iii) and vice versa (vi); identified by the Network-Based Statistics ($p_{FWE} < 0.05$). Each of the colour circles represents a state according to the colour scheme used in Supplementary Fig. 4. The diameter is scaled according to that state's averaged FO (panel a). Each arrow represents a transition.*

Supplementary Figure 6

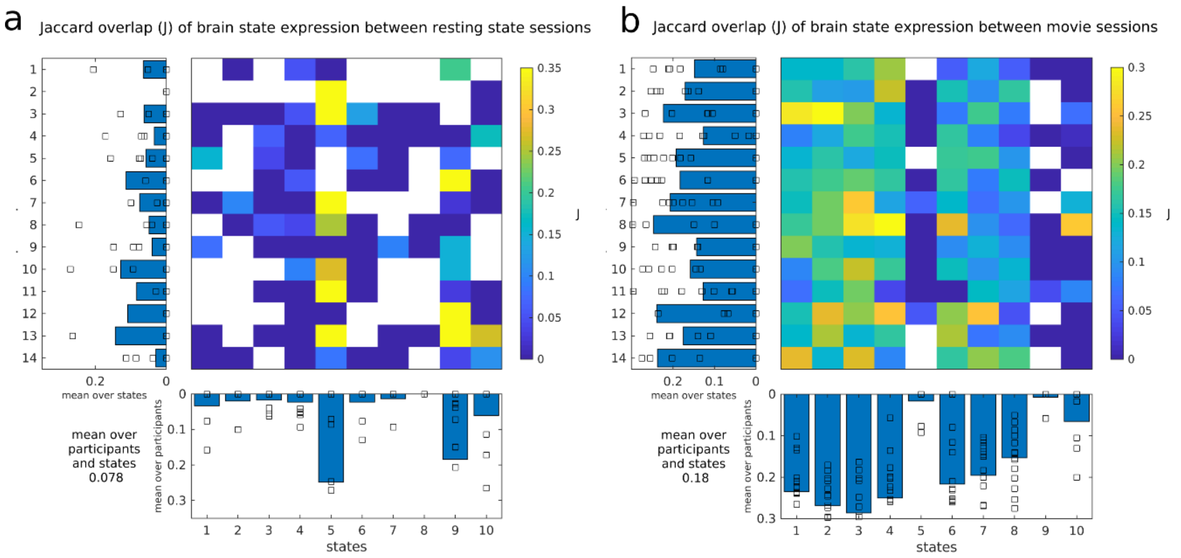

Supplementary Figure 6: Between-session consistency of brain state expression, calculated across participants and brain states. *We calculated the Jaccard overlap index (J) using the binarized brain state expression timeseries, for each combination of participant and brain state. If a participant had no expression of a brain state, it is not possible to calculate overlap and this data point was not further considered for calculating mean values; these instances are marked by white squares. Side panels indicate the mean overlap over participants (rows) and brain states (columns); individual values are plotted as black squares; n=14 participants examined over 2 sessions: rest session A and movie session A. (a) Jaccard overlap between resting state sessions. (b) Jaccard overlap between movie sessions.*

Supplementary Figure 7

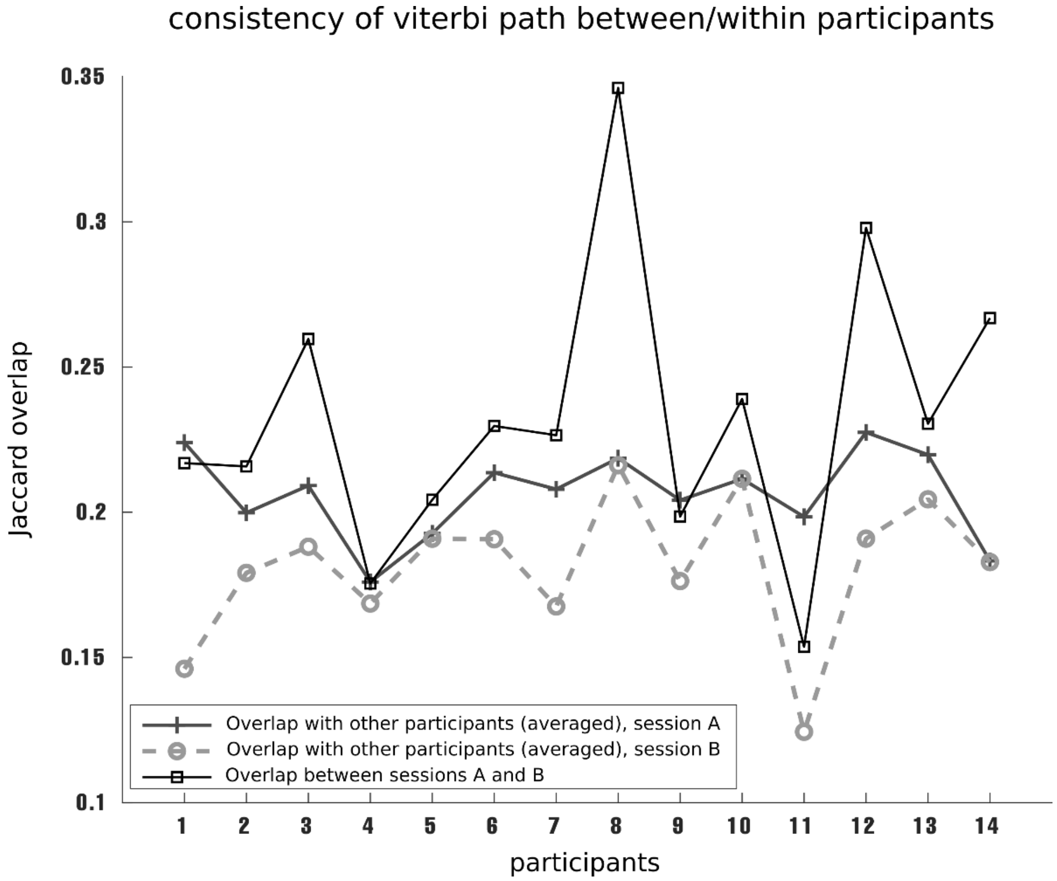

Supplementary Figure 7. Inter-session and inter-subjects consistency of each participant during movie viewing. *The inter-subject consistency was calculated using the Jaccard overlap between brain states expression (Viterbi path) averaged across subjects (subject's expression compared to the remaining 13 subjects). The inter-session consistency represents the Jaccard overlap between session A and B in the same subject. Within subject, inter-session consistency is higher than inter-subject consistency (paired two-sided t-test comparing across-subject (averaged over session A and B) and within-subject overlap (between session A and B),* t_{26} *= 2.85, p = 0.008; n=14 participants examined over 2 sessions: movie session A and movie session B).*

Supplementary Figure 8

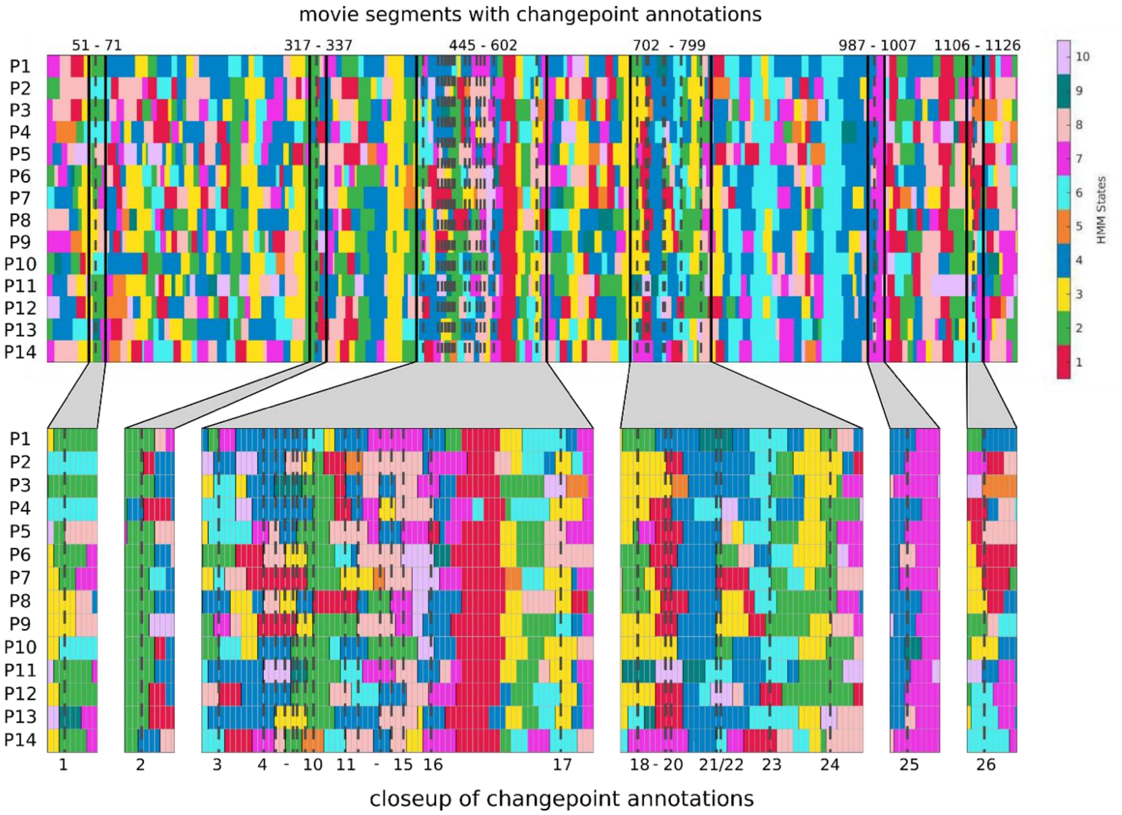

Supplementary Figure 8: Representation of the HMM brain states and the changepoints onsets (vertical black dashed lines) for session A (similar observation for session B). *The thin grey lines demarcate the temporal resolution of the MRI acquisition (TR = 2.2 seconds).*

Supplementary Figure 9

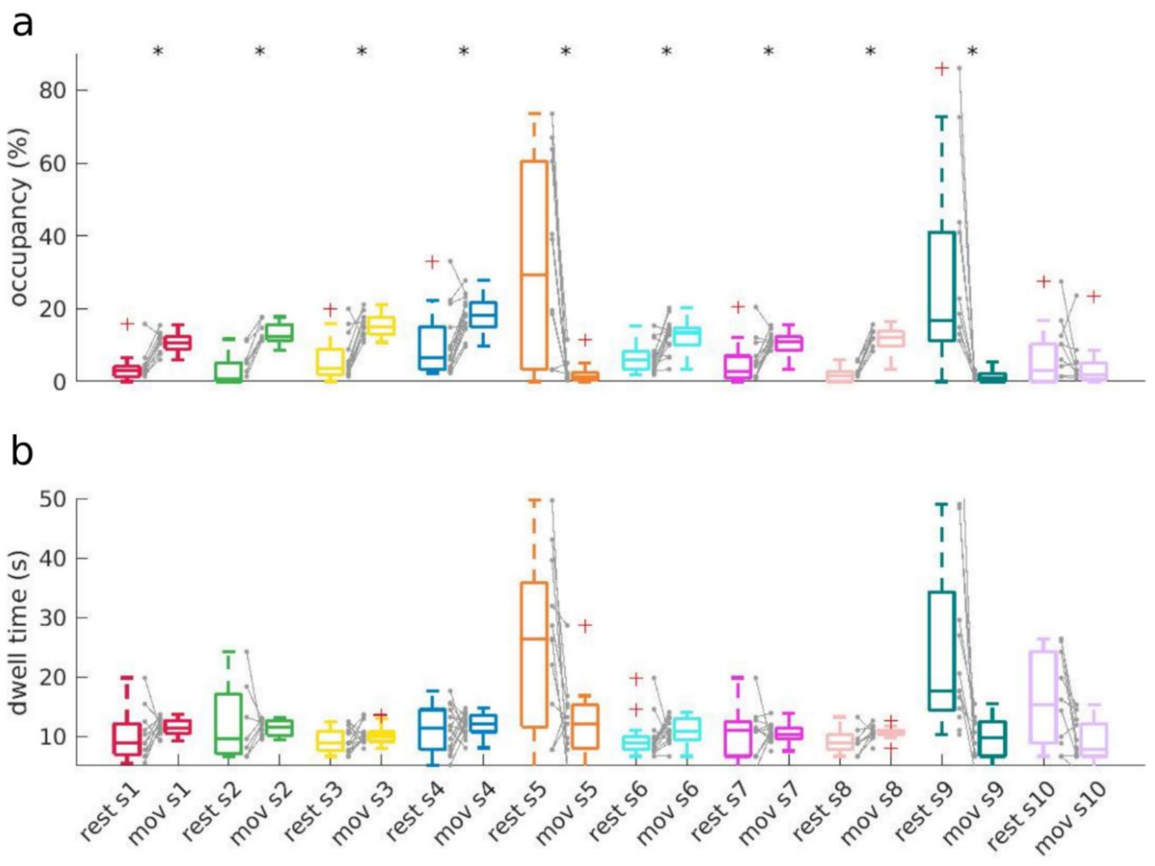

Supplementary Figure 9. Dynamic characteristics of brain states across rest and movie viewing during follow-up session (session B). *Boxplot data of N=14 participants. Asterisks indicate statistical significance (paired two-sided t-tests, $p < 0.05$; corrected for multiple comparison across 10 states; n=14 participants examined 2 consecutive (rest and movie) scans). Panel **a**: Fractional Occupancy. States 1-4 and 6-8 have higher occupancy in movie viewing, while the opposite is true for states 5 and 9. The exact p-values for states 1-10 are: $4.5e^{-6}$; $1.5e^{-7}$; $1.2e^{-4}$; $7.6e^{-4}$; $1.8e^{-3}$; $2.5e^{-3}$; $1.3e^{-3}$; $2.4e^{-7}$; $3.9e^{-3}$; 0.24. Panel **b**: State dwell times. Boxplots: upper (lower) box edge: 25th (75th) percentile; central line: median; dotted lines: 1.5 x interquartile length; whiskers extend to the most extreme data points not considered outliers; red plus: outliers.*

Supplementary Figure 10

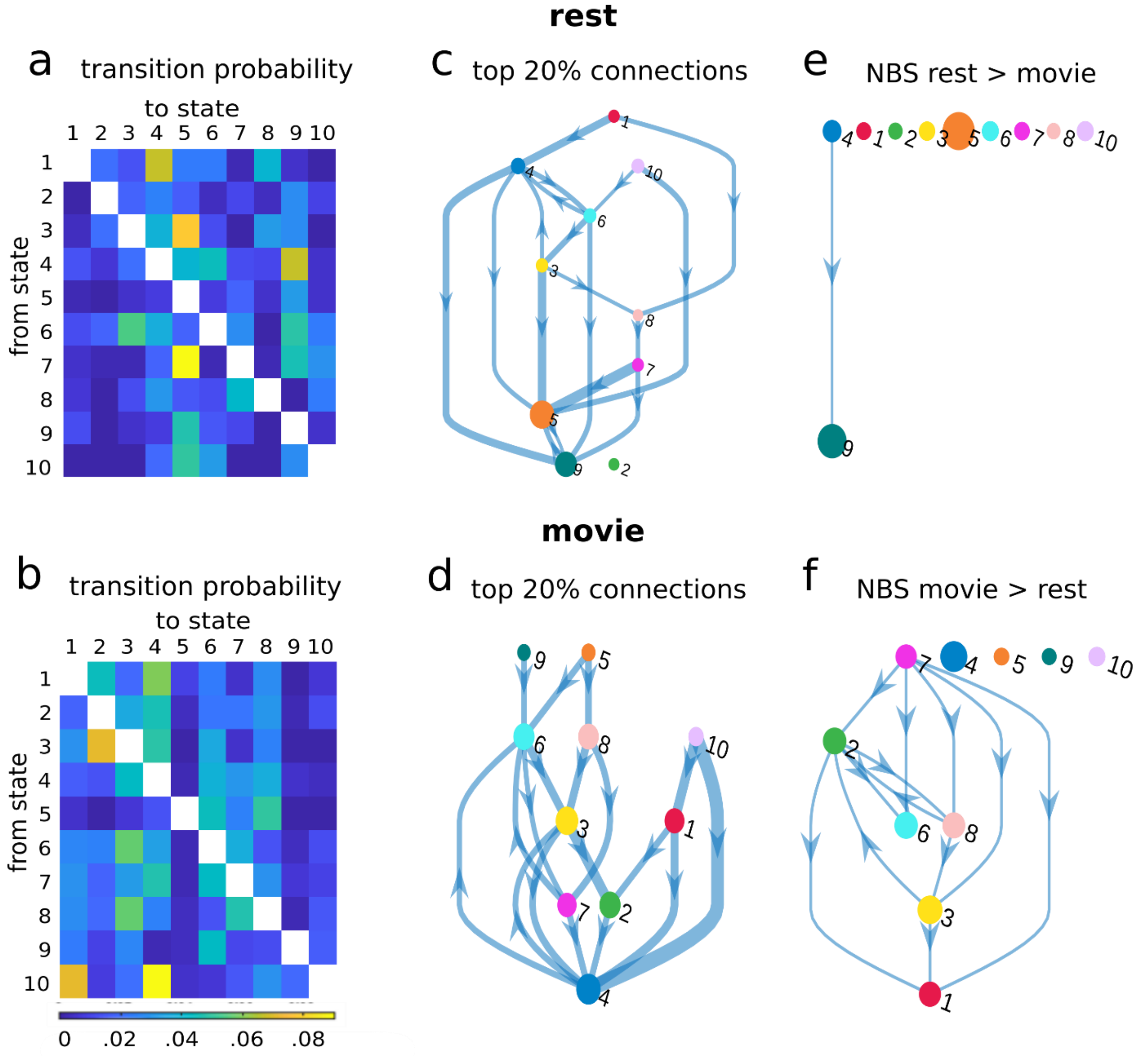

Supplementary Figure 10: Brain states transition probabilities during rest and movie viewing. *Data is averaged over N=14 participants, during session B (follow-up). Left column: Group averaged transition probability matrices for rest (**a**) and movie viewing (**b**). Diagonal elements were omitted for visualization. Middle column: Top 20% likely during rest (**c**) and movie viewing (**d**). Arrow thickness corresponds to group-averaged probability of that transition. Right column: State transitions having a significantly higher probability of occurring during rest than during movie viewing (**e**) and vice versa (**f**); identified by the Network-Based Statistics ($p_{NBS} < 0.05$). Each circle color represent a state according the color scheme used in **Fig. 1**. The diameter is scaled according to that state's fractional occupancy in Supplementary Fig. 2a. Each arrow represents a transition.*

Supplementary Figure 11

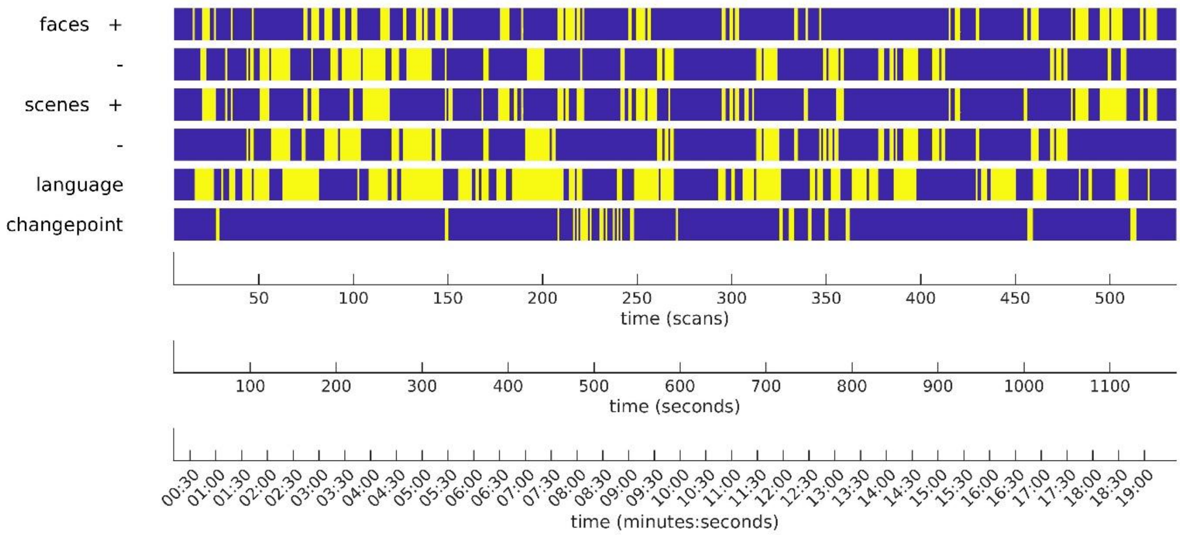

Supplementary Figure 11: Movie Annotations. *Occurrence of Positive/Negative Faces, Positive/Negative Scenes, Changepoints (a transition from one movie scene to another) and use of language, based on onset and offset times from the annotations made by S.S. The time scales are in scans (one scan = 2.2 seconds), in seconds and in minutes:seconds to facilitate inspection of the movie with a movie player.*

Supplementary Figure 12

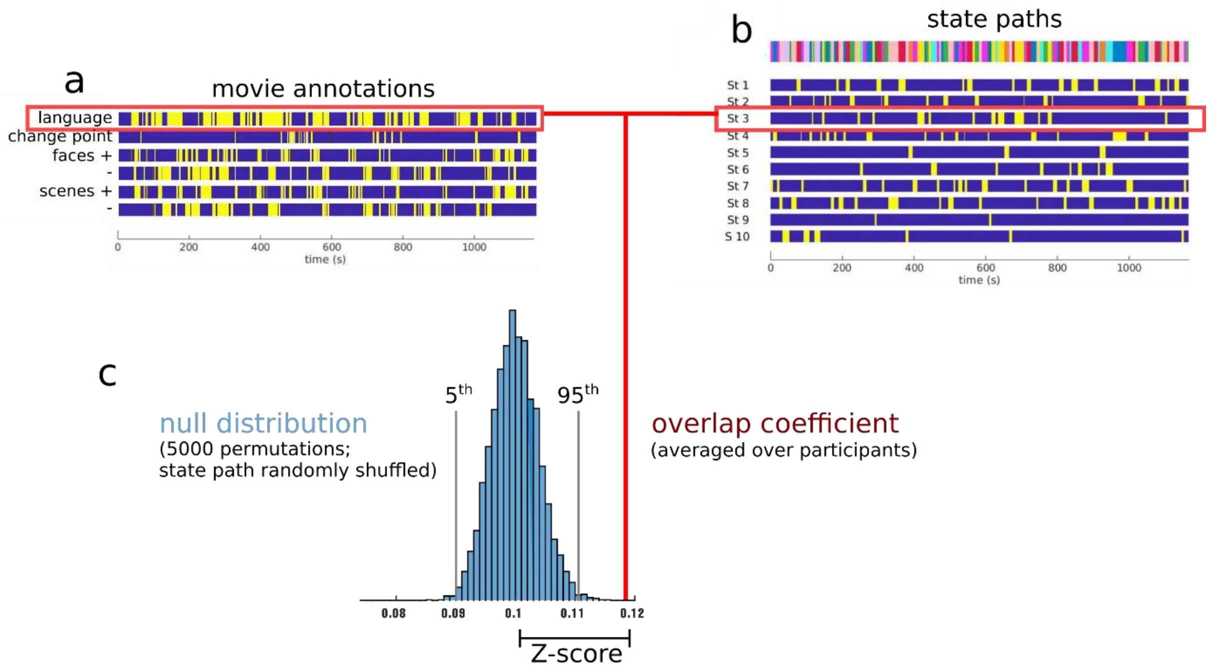

***Fig. 8. Association between brain states and movie annotations**. **a**: Binarised signals encoding for timing of movie annotations. **b**: Binarised signal describing if the brain is currently residing in that state. **c**: The (Szymkiewicz–Simpson) overlap index is calculated between the binarised signals from (a) and (b). This is compared to a null distribution generated by 5000 permutations. We report significant associations in Table 3 when $T > 3.16$, corresponding to $p_{FWE} < 0.05$ (60 comparisons).*

Supplementary Tables

Supplementary Table 1

Time (min:sec)	Plot Point	Scene description
**Act One**		
04:04	Catalyst: An inciting incident necessitates a major change for the protagonist	Will (the limbless man) is abused and humiliated at the sideshow, making him want to leave the carnival. Mendez (the Butterfly Circus showman) intervenes to help. The audience has a close view of Mendez approaching Will and Will's fearful and aversive reaction as the handheld camera executes a whip pan, pushing in to a close-up of Mendez as Will recoils.
04:11	Catalyst	Mendez calls Will "magnificent" but his actions and words are intrusive and the unexpectedly intimate approach is uninvited so Will recoils and spits at him, fearing ridicule. A medium close-up of Will, shot over Mendez's shoulder reveals the emotional force of this encounter.
04:37	Catalyst	An ugly close-up of the sideshow attendant shows him insulting Will, calling him a gimp. A close-up reaction shot of Will reveals that he is humiliated and angry.
04:50	Turning Point: establishes goal	Will realizes that he has just sabotaged his dream of escaping the sideshow and "joining one of those fancy shows."
05:03	Turning Point	Tattooed man: "You just spat on the showman from The Butterfly Circus. That was Mendez…"
05:14	Turning Point establishes narrative question	A tattooed sideshow worker laughs cruelly at Will and we observe Will's reaction in close-up as he realizes his predicament. This is an emotional low that establishes Will's goal. This is also the point at which the narrative question is formulated: How can Will achieve his goal of improving his life by joining a better circus show?
**Act Two**		

06:20	Development	The Butterfly Circus Strongman is startled when he discovers Will has stowed away in the truck. The audience sees a high angle mid-shot of Will lying in the truck with his back to the camera. Cheery background music plays on a gramophone when Mendez welcomes Will.
06:50	Development	Will meets a young boy called Sammy, who asks: "Where are your arms and legs?" and "will you be joining our show?" In this shot-reverse-shot dialogue sequence the camera looks down on Will slightly.
07:07	Obstacle (external)	Will discovers that he cannot attain his goal of joining a "fancy show" because The Butterfly Circus does not have a sideshow and Will has no skills to perform his own act.
07:18	Obstacle (external)	A low angle shot looking up at Mendez lends him authority as he asks Will why people visit sideshows. A close-up looking down on Will's face shows that he understands the cruelty of such shows and that his goal of being in such a show is problematic.
09:38	Obstacle (internal)	An African-American boy admires the Strongman's muscles and asks Will if he is also in the Butterfly Circus.
09:40	Obstacle (internal)	Will responds to the boy sadly, "No, not exactly". This establishes an internal obstacle that Will must overcome: he feels unworthy and useless.
09:56	Obstacle (internal)	Walking away the boy says he wants to be just like the Strongman; his father tells him that he can do anything if he wants to enough. Will's face reveals that he does not believe he can achieve greatness and be admired.
11:35	Turning Point: confronts internal obstacle	Mendez repeats humiliating taunts from the sideshow where Will used to work. Will is hurt and shocked, asking: "Why would you say that?"
11:50		Mendez: "Because you believe it." This emotional low point when Will faces his internal obstacle is accompanied by harsh dialogue, raised voices, close-ups of faces with tearful eyes and soft, sad violin music.

13:05	Turning Point: establishes new goal	Mendez: “You have an advantage. The greater the struggle, the more glorious the triumph.” Mendez is framed in a low-angle close-up that privileges him in the frame. Will’s new goal is to find his talent and triumph over adversity.
14:10	Confronts Internal Obstacle	Will falls while trying to cross the river and he feels helpless. As Will shouts for help his circus friends in the distance can’t hear him over the sound of the river.
14:20	Confronts Internal Obstacle	Mendez walks by. Will asks Mendez for help but Mendez says, “I think you’ll manage.” Melancholy violin music underscores this low point and the camera is also at ground level, showing Will helpless on the ground.
14:51	Overcomes Internal Obstacle	Will starts struggling to get up by himself, with determination. A hand-held shot from Will’s perspective shows him figuring out how to climb onto the log.
15:10	Overcomes Internal Obstacle	Will stands up on the log and cheers triumphantly, then begins to cross the river to the tune of upbeat fiddle and tambourine music.
15:34	Confronts External Obstacle	Will falls off the log into deep water. It looks as though Will is drowning. The audience hears muffled underwater bubble sounds and imagery as the music takes on a serious tone with a high anxious note and a low bowing sound.
15:46	Confronts External Obstacle	The circus folk notice Will is missing and search frantically for him in the water. Shots from the perspective of Will’s circus friends show the surface of the water dark and still.
16:20	Overcomes External Obstacle	Will overcomes his external obstacle and bobs to the surface: “Look! I can swim!”
**Act Three**		
17:05	Climax	Mendez tells the audience that Will is climbing 50 feet into the air and he will leap from a high platform and dive into a tiny water tank far below. Bird’s eye shot from Will’s point of view looking down at water from high diving board.

17:26	Climax	Will dives in and the crowd gasps.
17:37	Climax	Will then swims to the surface. Slow motion when Will surfaces and smiles.
17:38	Climax	Triumphant music and clapping.
18:11	Resolution	A disabled boy approaches Will after the show and hugs him. The boy's mother thanks him for being inspirational. From the perspective of the boy, the camera looks up to Will as the boy embraces him and he smiles, having found his talent and attained a sense of self-worth.

Supplementary Table 1: Timing and description of the 'plot twists' or narrative and emotional turning points in The Butterfly Circus. *This table summarizes the narrative analysis from an expert. The expert was trained in screenwriting and film theory and independently performed the narrative analysis to map the architecture of The Butterfly Circus. The narrative structure plots temporal progression against the emotional valence of the story, annotated with relevant observations about aesthetic techniques, dialogue, the protagonist's narrative trajectory, and emotional cues for the audience. These annotations are reproduced from earlier work*[5].

Supplementary References

1. Shirer, W. R., Ryali, S., Rykhlevskaia, E., Menon, V. & Greicius, M. D. Decoding subject-driven cognitive states with whole-brain connectivity patterns. *Cereb. Cortex N. Y. N 1991* **22**, 158–165 (2012).

2. Cousineau, D. Confidence intervals in within-subject designs: A simpler solution to Loftus and Masson's method. *Tutor. Quant. Methods Psychol.* **1**, (2005).

3. Bourgeois, F. & Lassalle, J.-C. An extension of the Munkres algorithm for the assignment problem to rectangular matrices. *Commun. ACM* **14**, 802–804 (1971).

4. Munkres, J. Algorithms for the assignment and transportation problems. *J. Soc. Ind. Appl. Math.* **5**, 32–38 (1957).

5. Nguyen, V. T. *et al.* Distinct Cerebellar Contributions to Cognitive-Perceptual Dynamics During Natural Viewing. *Cereb. Cortex* **27**, 5652–5662 (2017).
