# Supplementary material for: Movie viewing elicits rich and reliable brain state dynamics
Source: Nat Commun. 2020 Oct 5;11:5004. doi: 10.1038/s41467-020-18717-w (PMC7536385; doi:10.1038/s41467-020-18717-w)
Supplement: Supplementary file 3 — Reporting Summary [file 41467_2020_18717_MOESM3_ESM.pdf]

nature research

Corresponding author(s): Johan van der Meer; Luca Cocchi

Last updated by author(s): Aug 18, 2020

Reporting Summary

Nature Research wishes to improve the reproducibility of the work that we publish. This form provides structure for consistency and transparency in reporting. For further information on Nature Research policies, see our Editorial Policies and the Editorial Policy Checklist.

Statistics

For all statistical analyses, confirm that the following items are present in the figure legend, table legend, main text, or Methods section.

n/a	Confirmed	
☐	☒	The exact sample size (n) for each experimental group/condition, given as a discrete number and unit of measurement
☐	☒	A statement on whether measurements were taken from distinct samples or whether the same sample was measured repeatedly
☐	☒	The statistical test(s) used AND whether they are one- or two-sided<br>*Only common tests should be described solely by name; describe more complex techniques in the Methods section.*
☐	☒	A description of all covariates tested
☐	☒	A description of any assumptions or corrections, such as tests of normality and adjustment for multiple comparisons
☐	☒	A full description of the statistical parameters including central tendency (e.g. means) or other basic estimates (e.g. regression coefficient) AND variation (e.g. standard deviation) or associated estimates of uncertainty (e.g. confidence intervals)
☐	☒	For null hypothesis testing, the test statistic (e.g. F, t, r) with confidence intervals, effect sizes, degrees of freedom and P value noted<br>*Give P values as exact values whenever suitable.*
☐	☒	For Bayesian analysis, information on the choice of priors and Markov chain Monte Carlo settings
☐	☒	For hierarchical and complex designs, identification of the appropriate level for tests and full reporting of outcomes
☐	☒	Estimates of effect sizes (e.g. Cohen's d, Pearson's r), indicating how they were calculated

Our web collection on statistics for biologists contains articles on many of the points above.

Software and code

Policy information about availability of computer code

Data collection	The movie stimulus was programmed in Presentation version 16.3 (Neurobehavioral Systems, Inc.).
Data analysis	Image preprocessing was performed using fMRIPprep version 1.1.5 and the Python toolbox Nilearn version 0.5.0.<br><br>Heart rate signal was retrieved using FMRIB FastR version 1.21 and the Tapas IO Toolbox version 2016 was used to create heart rate (HR) in the same temporal resolution as the fMRI timeseries.<br><br>Pupil diameter preprocessing was done using custom MATLAB scripts, made available in our Github repository: https://github.com/brain-modelling-group/MovieBrainDynamics.<br><br>The HMM-MAR MATLAB toolbox (https://github.com/OHBA-analysis/HMM-MAR; commit version 7a5915c) was used to perform Variational Bayes inference on the HMM.<br><br>Reverse inference of HMM brain states was performed using Neurosynth software provided from the Github repository at: https://github.com/neurosynth/neurosynth; commit version 948ce7.<br><br>Network-Based Statistics (NBS) version 1.2 was applied to reveal the network of state transitions that are significantly more expressed during movie watching compared to rest (https://www.nitrc.org/projects/nbs/).

For manuscripts utilizing custom algorithms or software that are central to the research but not yet described in published literature, software must be made available to editors and reviewers. We strongly encourage code deposition in a community repository (e.g. GitHub). See the Nature Research guidelines for submitting code & software for further information.

Data

Policy information about availability of data

All manuscripts must include a data availability statement. This statement should provide the following information, where applicable:

- Accession codes, unique identifiers, or web links for publicly available datasets
- A list of figures that have associated raw data
- A description of any restrictions on data availability

The source data belonging to the figures in the main manuscript is stored in an Excel file (SourceData.xls). This file is provided as suppelementary material, and is also stored in the "figure_sourcedata" folder of our github repository.
- Data in the form of exctracted timeseries used to generate figures are available in the "data" folder of our github repository here: https://github.com/brain-modelling-group/MovieBrainDynamics.
The Neurosynth database is available here: www.neurosynth.org. It is also accessible using the python interface mentioned in the Software section.

Field-specific reporting

Please select the one below that is the best fit for your research. If you are not sure, read the appropriate sections before making your selection.

☒ Life sciences ☐ Behavioural & social sciences ☐ Ecological, evolutionary & environmental sciences

For a reference copy of the document with all sections, see nature.com/documents/nr-reporting-summary-flat.pdf

Life sciences study design

All studies must disclose on these points even when the disclosure is negative.

Sample size	Previously published analyses of the current neuroimaging data showed that the sample size was sufficient to detect meaningful and significant changes in patterns of fMRI signals related to perception of movie scenes (Nguyen et al., Cer. Cortex, 2017).
Data exclusions	Three participants were excluded because of excessive in-scanner head motion (>10% of volumes with a frame-wise displacement greater than 0.4 mm). Thus, rest and movie data from 18 participants were included at the baseline session. 14 participants completed both sessions (i.e. baseline and 3-months follow-up).
Replication	Results have been replicated once (with 17 out of 21 participants, 81%) using a second within-subject experiment (performed 3 months after the baseline experimental session). Replication was succesful.
Randomization	This is not a randomized treatment study.
Blinding	Blinding is not relevant to this study because it is not a randomized treatment study.

Reporting for specific materials, systems and methods

We require information from authors about some types of materials, experimental systems and methods used in many studies. Here, indicate whether each material, system or method listed is relevant to your study. If you are not sure if a list item applies to your research, read the appropriate section before selecting a response.

Materials & experimental systems

n/a	Involved in the study	
☒	☐	Antibodies
☒	☐	Eukaryotic cell lines
☒	☐	Palaeontology and archaeology
☒	☐	Animals and other organisms
☐	☒	Human research participants
☒	☐	Clinical data
☒	☐	Dual use research of concern

Methods

n/a	Involved in the study	
☒	☐	ChIP-seq
☒	☐	Flow cytometry
☐	☒	MRI-based neuroimaging

Human research participants

Policy information about studies involving human research participants

Population characteristics	Twenty-one healthy participants (11 females, 10 males; right-handed; 21-31 years, mean age 27 ± 2.7 years) were recruited for this study.
Recruitment	The participants were recruited from the University of Queensland by advertisement and provided written informed consent. All participants received monetary compensation for their participation in the study ($50). The study includes a sample of healthy, tertiary-educated, young adults that were naïve to the study's material and objectives. The sample was chosen by convenience and is representative with regard to the purpose of the research.
Ethics oversight	The study was approved by the human ethics research committee of the University of Queensland and was conducted according to National Health and Medical Research Council guidelines. Written consent was obtained for all participants.

Note that full information on the approval of the study protocol must also be provided in the manuscript.

Magnetic resonance imaging

Experimental design

Design type	Resting state and movie viewing
Design specifications	For each session, fMRI data were acquired from participants during an 8-minute (eyes closed) resting-state session, followed by viewing of a 20-min short movie "The Butterfly Circus". The Butterfly Circus narrates an intense, emotionally evocative story of a man born without limbs who is encouraged by the showman of a renowned circus to overcome obstacles of self-worth and reach his own potential. The narrative architecture of The Butterfly Circus map onto three distinctive drama acts with significant developments, complications and turning points for each act (Supplementary Table 1). Moreover, the following basic annotations had been drafted in previous work (Nguyen, V. T. et al. Cer. Cortex, 2017): (i) the use of language, (ii) change of scenes, (iii-vi) Positive/Negative Faces and (vi-viii) Positive/Negative Scenes (details in Supplementary Fig. 11).
Behavioral performance measures	After the movie, the participants completed a questionnaire that assessed their impression of the movie (see Methods).

Acquisition

Imaging type(s)	fMRI and structural (T1)
Field strength	3T
Sequence & imaging parameters	Data were acquired using a Siemens TIM Trio scanner equipped with a 12-channel head coil. Functional and structural images were acquired from a whole-body 3-Tesla Siemens Trio MRI scanner equipped with a 12-channel head coil (Siemens Medical System, Germany). Functional images were acquired using a single-shot gradient-echo Echo Planar-Imaging (EPI) sequence with the following parameters: repetition time (TR) 2200 ms, echo time (TE) 30 ms, flip angle (FA) 79°, field of view (FOV) 192×192 mm, pixel band-width 2003 Hz, a 64×64 acquisition matrix, 44 axial slices, and 3×3×3 mm voxel resolution. A high-resolution T1-weighted MPRAGE structural image covering the entire brain was also acquired from each participant with the following parameters: TE=2.89 ms, TR=4000 ms, FA=9°, FOV=240×256 mm, and voxel size 1×1×1 mm.
Area of acquisition	Whole brain scan.

Diffusion MRI ☐ Used ☒ Not used

Preprocessing

Preprocessing software	fMRIPprep version 1.1.5
Normalization	Spatial normalization to standard space was performed using ANTs (antsRegistration; fMRIPrep) in a multiscale, mutual-information based, nonlinear registration scheme.
Normalization template	Images were spatially normalized to the MNI152NLin2009cAsym template.
Noise and artifact removal	Structural images were first corrected for intensity non-uniformity and spatially normalized to MNI space (ICBM 152 Nonlinear Asymmetrical template version 2009c). Brain tissue segmentation of cerebrospinal fluid (CSF), white-matter (WM) and gray-matter (GM) was also performed. Functional images were slice-time corrected, motion corrected, co-registered to the structural image, normalized to MNI space, and spatially smoothed with a 6 mm Gaussian kernel. ICA-AROMA was subsequently performed using "non-aggressive" denoising. Two confounding time-series were obtained from functional image preprocessing: global signal in white matter (WM), and global signal in the cerebrospinal fluid (CSF). After spatial preprocessing, temporal preprocessing was performed with the toolbox Nilearn. This consisted of filtering the data between 0.01 and 0.15 Hz to capture neural signal associated to both rest and task and regression of global WM and CSF signals.
Volume censoring	Not applied

Statistical modeling & inference

Model type and settings	The HMM-MAR MATLAB toolbox (https://github.com/OHBA-analysis/HMM-MAR) was used to perform Variational Bayes inference on the Hidden Markov Model (HMM), using 500 training cycles. The total number of states needs to be specified a priori. Previous studies modelling functional MRI dynamics in healthy individuals considered between 5 and 12 states. In our main analysis the HMM input is a 20,860 (14 participants with each 1,490 timepoints) by 14 (average signal from 14 network masks) matrix. We used Model fit and the Akaike Information Criterion (AIC) metrics to infer the HMM with 10 states. In addition, we inferred the HMM also with 6, 8, 12, 14 and 25 states, with each state choice decoded 15 times. We found that using 12 states or more yielded HMM results in which several states were not occupied. Therefore, the use of 12 or more states likely reached a practical limit on the HMM on our current dataset.
Effect(s) tested	1. Spatial definition of the 14 canonical brain networks<br>The HMM was used to model the temporal dynamics of 14 canonical brain networks (BNs) during the first and second movie viewing session, as well as the first and second rest condition. The spatial extent of these BNs were defined according to an established reference definition that maximally disambiguates cognitive states (Shirer et al., 2012; Supplementary Fig. 1).<br><br>2. Functional relevance of HMM brain states<br>The Neurosynth database contains nearly 14,300 fMRI studies and 507,000 reports of BOLD-inferred activity (www.neurosynth.org). By mapping keywords (topics) extracted from the literature body to the locations of the activity, it enables broad cognitive functions/states to be decoded from brain activity in entire studies or individual participants. The decoding itself is an association test: it reflects the probability of a psychological process being present given the pattern of activity over several regions in the brain. The Neurosynth framework provides a comprehensive set of whole-brain term-to-activation 'topic' maps that allow one to calculate either forward associations (P(Activation|State)) or reverse associations ((P (State|Activation)). In the present study, we associate the brain state of our HMM model to the topic maps of 16 general terms chosen to encompass a variety of brain processes that apply to movie viewing. We correlated the spatial distribution of each brain state to the topic maps, effectively decoding the range of mental states that are associated with each brain state during watching of the Butterfly Circus movie. Decoding was performed using a python notebook obtained from the Neurosynth github webpage (https://github.com/neurosynth/neurosynth).<br><br>3. Reproducibility of HMM state paths over participants and sessions<br>In order to calculate between-participant consistency of state paths, we calculated consistency in a sliding window of 9 consecutive BOLD volumes (19.8 seconds). Within this window, for each of the 10 brain states, we counted the number of participants that had this state expressed at least once and identified the most frequently expressed state. We then contrasted the states expressed during movie watching with those of the resting state scans. In order to calculate the consistency of brain states over sessions, for each state and each participant, we constructed a binary vector of 0 (brain state not expressed) and 1 (brain state is expressed) for each time-point during movie viewing and rest, for session A and session B. To assess consistency over sessions (A and B), we calculated the Jaccard Overlap index between the binary vectors for each brain state, and averaged them. A two tailed paired T-test was performed on these values to test whether movie viewing had more consistent brain-state overlap than resting state.<br><br>4. Comparison of brain states dynamics between rest and movie watching<br>In order to compare brain state dynamics between rest and movie watching, we computed the dynamic metrics (FO and state dwell time) for each brain state. Significant differences between these conditions were assessed with two-tailed paired t-tests ($p < 0.05$, family-wise corrected). We also compared the state transition probabilities (averaged over participants) between the conditions. NBS was applied to reveal the network of state transitions that are significantly more expressed during movie watching compared to rest, and conversely during resting state compared to movie viewing (right column).<br><br>5. Association between brain state and electrophysiological data<br>The heart rate and pupil diameter values were segmented according to the brain state path and averaged across time to calculate (for each brain state) the value during a visit to brain states 1-10. Following, across the entire movie averages were calculated for heart rate and pupil diameter to provide a baseline, which was subtracted from the brain state-specific value to generate the deviation value for heart rate (delta HR) and pupil diameter (delta PD). This produced participant-specific deviation values. A one-sample T-test was then used to assess the likelihood of the observed deviations against zero deviation.<br><br>6. Association between brain state and movie annotation data<br>To obtain information about the putative connection between HMM brain states and movie annotations reflecting the unfolding narrative, we calculated the Szymkiewicz–Simpson overlap between two 'state' vectors. The HMM brain state vector is composed of values 1 and 0 denoting the expression or lack of an expression respectively of the the HMM brain state. The story annotations are also converted to vectors of 0 and 1 according to their onset and offset times. Annotations vectors were generated for Positive Faces, Negative Faces, Positive Scenes, Negative Scenes, Language and Changepoint. In order to test significance, the overlap index was compared to a null distribution of overlap indices generated by 5000 permutations of the brain state vectors and their overlaps with the annotation vector. For each iteration, the movie annotation vector was randomly shuffled, the overlap between HMM state vector and annotation vector was (re-)calculated, and finally averaged over participants to create a new random value. The permutations form a null distribution allows for a comparison with the observed overlap index to infer statistical significance with a t-score.<br><br>7. Post-movie questionnaire<br>The post-movie questionnaire had 8 questions. Four questions were omitted for Representation Similarity Analysis (RSA) analysis due to the lack of variability across participants. To quantify participant differences in movie evaluation, a multidimensional scaling approach was applied to plot the questionnaire answers as points in a 2-D representation (engagement by evoked emotion).

8. Association between movie ratings and brain state dynamics

We used the inter-subject representational similarity analysis (IS-RSA) to assess how brain and behavioural data are represented in the group sample. Three inter-subject distance matrices were constructed for representations of brain dynamics (FO and state transition) and movie impressions. To calculate inter-subject distances for movie impression the Euclidean distance of questionnaire ratings between each possible pair of participants was measured, producing an 18 (participants) x 17 matrix. For the FO representation, for every possible pair of participants, the correlation between the 10 FO values (one for each state) was calculated to produce the inter-subject distance matrix. Silimarly, for the State Transition representation, for every possible pair of participants, the correlation between the State Transition matrices (10 states x 9 transitions to another state) was calculated to produce the inter-subject distance matrix. In this way we generated a single representation for movie impressions and two representations for brain states dynamics (one for FO and one for state transitions). To assess the strength of associations between the brain states and movie rating representations, we calculated a Pearson's correlation between the lower triangular parts of their corresponding matrices. Statistical significance was assessed using permutations. From the null distribution, Z scores (and associated p-values) were obtained.

Specify type of analysis: ☐ Whole brain ☒ ROI-based ☐ Both

Anatomical location(s): Canonical whole-brain resting-state networks (14 networks)

Statistic type for inference (See Eklund et al. 2016): Not applicable

Correction: Family-Wise Error rate correction.

Models & analysis

n/a	Involved in the study
☒	☐ Functional and/or effective connectivity
☐	☒ Graph analysis
☐	☒ Multivariate modeling or predictive analysis

Graph analysis: We did not use a standardized Graph analysis approach, with matrices whose elements represent connectivity. In our case, matrix elements represented the transition probability between 10 distinct brain states obtained from the HMM analysis on concatenated movie and rest data. For each participant, matrices were formed for movie viewing and resting state separately from the corresponding brain state paths. Network Based Statistics (NBS) analysis was performed to obtain sets of state transitions (i.e. graph edges) which occurred more during movie viewing than during resting state and vice versa. No global or nodal properties were analyzed.

Multivariate modeling and predictive analysis: hidden Markov model (HMM)
